# Tautomerization, molecular structure, transition state structure, and vibrational spectra of 2-aminopyridines: a combined computational and experimental study

**DOI:** 10.1186/s40064-015-1363-2

**Published:** 2015-10-09

**Authors:** Jamelah S. Al-Otaibi

**Affiliations:** Department of Chemistry, College of Science, Princess Nourah bint Abdulrahman University, Riyadh, 11951 Saudi Arabia

**Keywords:** Vibrational analysis, Tautomerization, DFT, Transition state, 2-amino-methylpyridine

## Abstract

**Background:**

2-amino pyridine derivatives have attracted considerable interest because they are useful precursors for the synthesis of a variety of heterocyclic compounds possessing a medicinal value. In this work we aim to study both structural and electronic as well as high quality vibrational spectra for 2-amino-3-methylpyridine (2A3MP) and 2-amino-4-methylpyridine (2A4MP).

**Results:**

Møller–Plesset perturbation theory (MP2/6-31G(d) and MP2/6-31++G(d,p) methods were used to investigate the structure and vibrational analysis of (2A3MP) and (2A4MP). Tautomerization of 2A4MP was investigated by Density Functional Theory (DFT/B3LYP) method in the gas phase. For the first time, all tautomers including NH → NH conversions as well as those usually omitted, NH → CH and CH → CH, were considered. The canonical structure (2A4MP1) is the most stable tautomer. It is 13.60 kcal/mole more stable than the next (2A4MP2). Transition state structures of pyramidal N inversion and proton transfer were computed at B3LYP/6-311++G(d,p). Barrier to transition state of hydrogen proton transfer is calculated as 44.81 kcal/mol. Transition state activation energy of pyramidal inversion at amino N is found to be 0.41 kcal/mol using the above method. Bond order and natural atomic charges were also calculated at the same level. The raman and FT-IR spectra of (2A3MP) and (2A4MP) were measured (4000–400 cm^−1^). The optimized molecular geometries, frequencies and vibrational bands intensity were calculated at ab initio (MP2) and DFT(B3LYP) levels of theory with 6-31G(d), 6-31++G(d,p) and 6-311++G(d,p) basis sets. The vibrational frequencies were compared with experimentally measured FT-IR and FT-Raman spectra.

**Conclusion:**

Reconsidering the vibrational analysis of (2A3MP) and (2A4MP) with more accurate FT-IR machine and highly accurate animation programs result in new improved vibrational assignments. Sophisticated quantum mechanics methods enable studying the transition state structure for different chemical systems.

**Electronic supplementary material:**

The online version of this article (doi:10.1186/s40064-015-1363-2) contains supplementary material, which is available to authorized users.

## Background

Pyridine and its derivatives has been the subject of investigation by several workers in the last 50 years. The structural and vibrational spectrum of pyridine and its derivatives have been extensively studied and analyzed in recent years because of their involvement in bioactivities and applications in pharmaceutical, agro chemical and many other industries (Jose and Mohan 2006; Othmer 1997; Pierrat et al. 2005). In particular it has been mentioned that 2-amino pyridine derivatives have attracted considerable interest because they are useful precursors for the synthesis of a variety of heterocyclic compounds possessing a medicinal value (Temple et al. 1987; Janssens et al. 1985; Mantlo et al. 1991; Oguchiet al. 2000). It has been reported recently that the inhibitory properties of 3-nitropyridine derivatives and their salts can be used as therapeutic or preventive agents for hepatitis B and acquired immune deficiency syndrome (AIDS) (Yoon et al. 2004). Various spectroscopic studies have been reported on methyl substituted pyridine derivatives which have cholesterol lowering properties and anti–cancer activity (Suheyla Kurkcuoglu et al. 2009; Green et al. 1963; Long and George 1963). The pyridine ring appears in a large number of natural substances such as vitamin B5, vitamin B6, pyridoxal and pyridoxamine; and drugs such as nifedipine, nichetamine and sulphapyridine (Ög˘retir et al. 2006; Ziessel 2001; Hagadorn et al. 2000; Holland et al. 2001; Marlin et al. 2001; Lipinski et al. 2001). The spectroscopic studies of N–heterocyclic molecules including substituted pyridines have become quite interesting as they are the constituents of DNA and RNA (Arivazhagan and Krishnakumar 2003; Singh and Srivastava 2009; Yadav et al. 2007). Also Pyridine and its derivatives have been studied as corrosion inhibitors (Arshadi et al. 2004; Yadav and Wadhwani 1993; Talati and Gandhi 1983; Yurt et al. 2005; Yurt et al. 2006; Ashassi-Sorkhabi et al. 2006; Lashkari and Arshadi 2004; Xiao-Ci et al. 2000).

In the literature, there are many studies reporting on the vibrational analysis of pyridine (Pongor et al. 1984; Stidham and Dilella 1979; Stidham and Dilella 1980; Dilella and Stidham 1980; Dilella 1980; Zerbi et al. 1963) and its methylated derivatives (Draeger 1983). X-ray, IR and Raman studies as well as quantum chemical calculations performed for 3 and 5-nitroderivatives of 2A4MP (Bryndal et al. 2012). DFT and ab initio computation of structure and vibrational frequencies of pyridine and its isotopomers were reported (Szafran and Koput 2001).

The geometry of isomeric pyridines were studied by X-ray crystallography (Kubiak et al. 2002), microwave spectroscopy (Ford 1975) and surface-enhanced Raman spectroscopy (Arenas et al. 1993). DFT vibrational studies of 5-bromo-2-nitropyridine (Sundaraganesan et al. 2005), pyridinium complexes (Tayyari et al. 2010), and 2-chloro-5-bromopyridine (Hiremath et al. 2010; Kishor and Bhoop 2013) were reported. Tautomerization and rotamerization of aminopyridines has been reported (Davoodnia et al. 2011; Alkorta and Elguero 2002; Pietrzycki et al. 1993). The vibration (IR and Raman) spectra of aminopyridines, methyl-substituted aminopyridines and hydrogen chlorides of aminopyridines had been determined experimentally by Spinner (Spinner 1962). However, to the best of our knowledge, according to literature survey there is no results based on quantum chemical calculations, on tautomerization, transition state structures and FT-IR/FT-Raman spectral studies on (2A3MP) and (2A4MP). Hence in the present work, we reported detailed tautomeization, transition state activation energies and interpretations of the infrared and Raman spectra based on the experimental and theoretical results. Reconsidering the vibrational analysis of (2A3MP) and (2A4MP) with more accurate FT-IR machine and highly accurate simulation programs lead to new improved vibrational assignments.

## Results and discussion

### Geometrical features

**Fig. 1:**
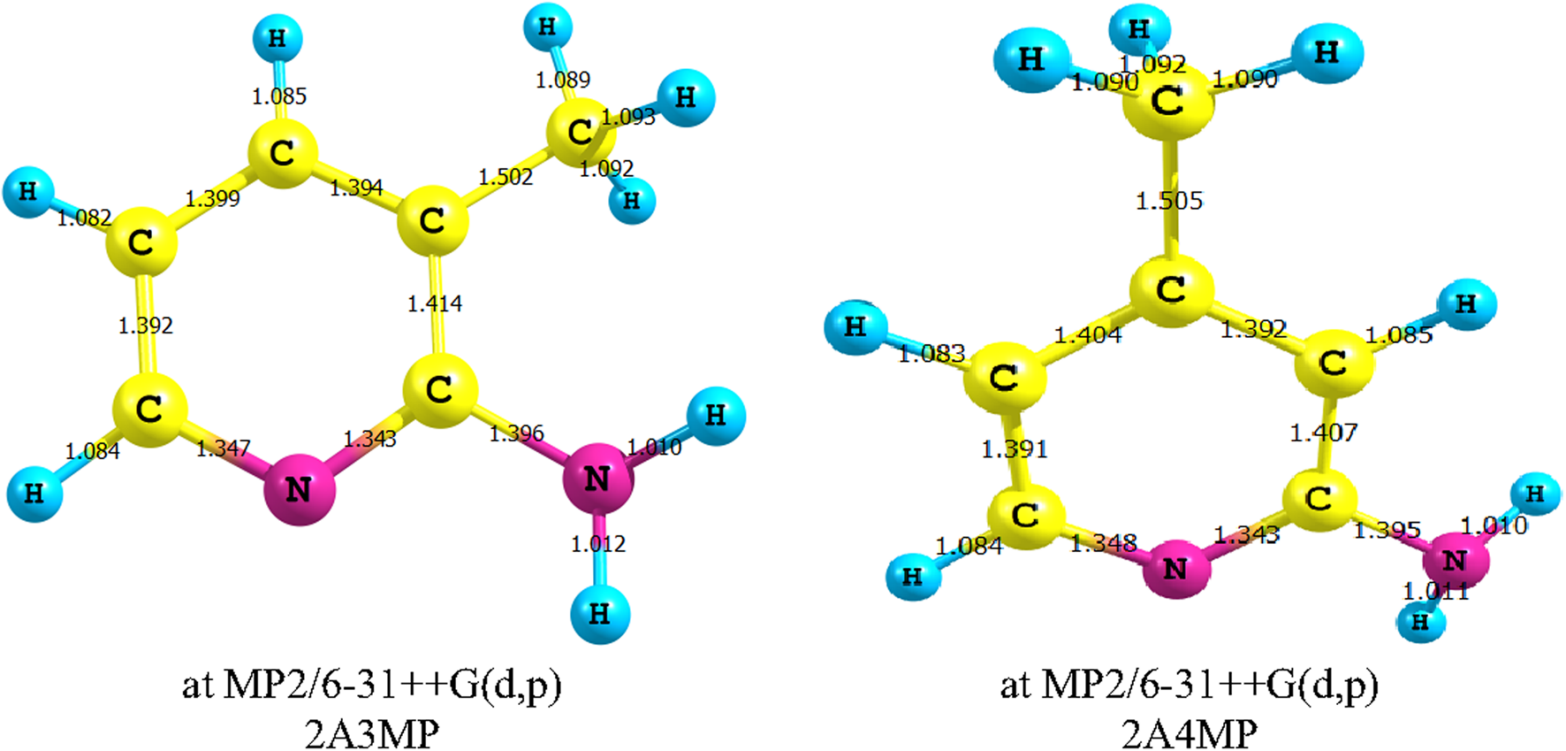
Optimized geometry at MP2/6-31++G(d,p) showing all bond lengths

The optimized molecular structures of 2A3MP and 2A4MP are obtained at different computational methods. The optimized structural parameters were shown in Additional file 1: Tables S1 and S2 for 2A3MP and 2A4MP in the Additional files respectively. By allowing the relaxation of all parameters, the calculations converge to optimized geometries, which correspond to true energy minima, as revealed by the lack of imaginary frequencies in the vibrational mode calculation. These molecules have three CN, two NH, five CC and six CH bonds. It was reported that the HF approximation is, in general, insufficient to study the geometry of amino group-containing compounds (Sponer and Hobza 1994a, b, c; Sponer et al. 1996). Thus MP2 with high basis sets was used to examine the molecular structure and amino group pyramidalization of 2A3MP and 2A4MP. The geometrical parameters at the MP2 methods are shown in Fig. 1 and in Additional file 1: Tables S3 and S4 for 2A3MP and 2A4MP respectively. As shown in Fig. 1, the C-N_py_ bond lengths are slightly different. The one closer to the amino group is shorter by a value of 0.004 and 0.005 Å for 2A3MP and 2A4MP respectively as predicted by MP2/6-31++G(d,p). This could be due to the interaction of the amino nitrogen lone pair with the C-N_py_ bond. This interaction leads to the increase of double bond character of the C-N_py_ bond and hence the shortening of the bond closer to the amino group. All methods used throughout this work prove the pyramidalization of the amino group as displayed in Additional file 1: Tables S1–S4. However, the inclusion of d-polarization functions in the amino group hydrogen is quite essential. The non-planarity of the amino group was mostly found to be much larger at the MP2 level, compared with the uncorrelated methods. Computations reveals the asymmetry of the amino group hydrogen dihedral angles, which is due to the repulsive electrostatic interactions with the neighboring hydrogens. The calculations revealed a significant difference between the correlated and uncorrelated results. It is easily noticed from Additional file 1: Tables S1–S4 that the B3LYP/6 311++G(d,p) method overestimates bond lengths, particularly the CH bonds. The CC bond distances are differing in value. The reasons for that may be the replacement of hydrogen atoms in the pyridine ring by methyl groups and the distortion of the aromatic ring hexagonal symmetry in the pyridine ring. Geometry based on MP2 and B3LYP calculation shows that the average bond lengths of CC and CH in the pyridine ring are 1.394 and 0.990 Å, for 2A3MP and 2A4MP. Comparing MP2 and B3LYP methods, the predicted N–H (amino group) bond lengths are found at 1.011 and 1.009 Å for 2A3MP and 2A4MP, respectively. The experimental value (1.001 Å), is more closer to that predicted by B3LYP method (Fukuyo et al. 1982). The optimized CN bond length is 1.347 and 1.340 Å for 2A3MP and 1.348 and 1.342 Å for 2A4MP by MP2 and B3LYP methods, respectively. Both methods predict the C–N bond length very close to each other but shorter than the measured value (1.402 Å measured for a similar aromatic ring, aniline) (Fukuyo et al. 1982). Some calculated ring angles, C2–C1–C6 and C5–C6–C1 are deviating from the perfect hexagon value (120°) at C1 and C6 atom positions for 2A3MP and the same deviation is also shown at the corresponding atom positions, C2 and C6 for 2A4MP. This could be due to the substitutions of –NH_2_ and –CH_3_ groups. This effect results from the interaction of the N lone pair of electrons with the aromatic ring.

The B3LYP geometry is similar to the MP2 geometry at the same basis set. The bond distance C2–C3 in 2A3MP (1.399 Å) at MP2/6-31++G(d,p) is similar to that (1.400 Å) at B3LYP/6-31G(d,p) and the bond distance C3–C4 is the same as predicted by the two methods (1.392 Å). The effect of adding diffuse functions and polarization functions on H atoms on the predicted geometry by the energy correlated method MP2 could be observed in Additional file 1: Tables S3 and S4. The increase of the basis set size overestimates the ring C–C and C–N bond distances and underestimates the C–H distances. The exo-cyclic C–C and C–N bonds remain unchanged.

### Tautomerization of 2A4MP

**Fig. 2:**
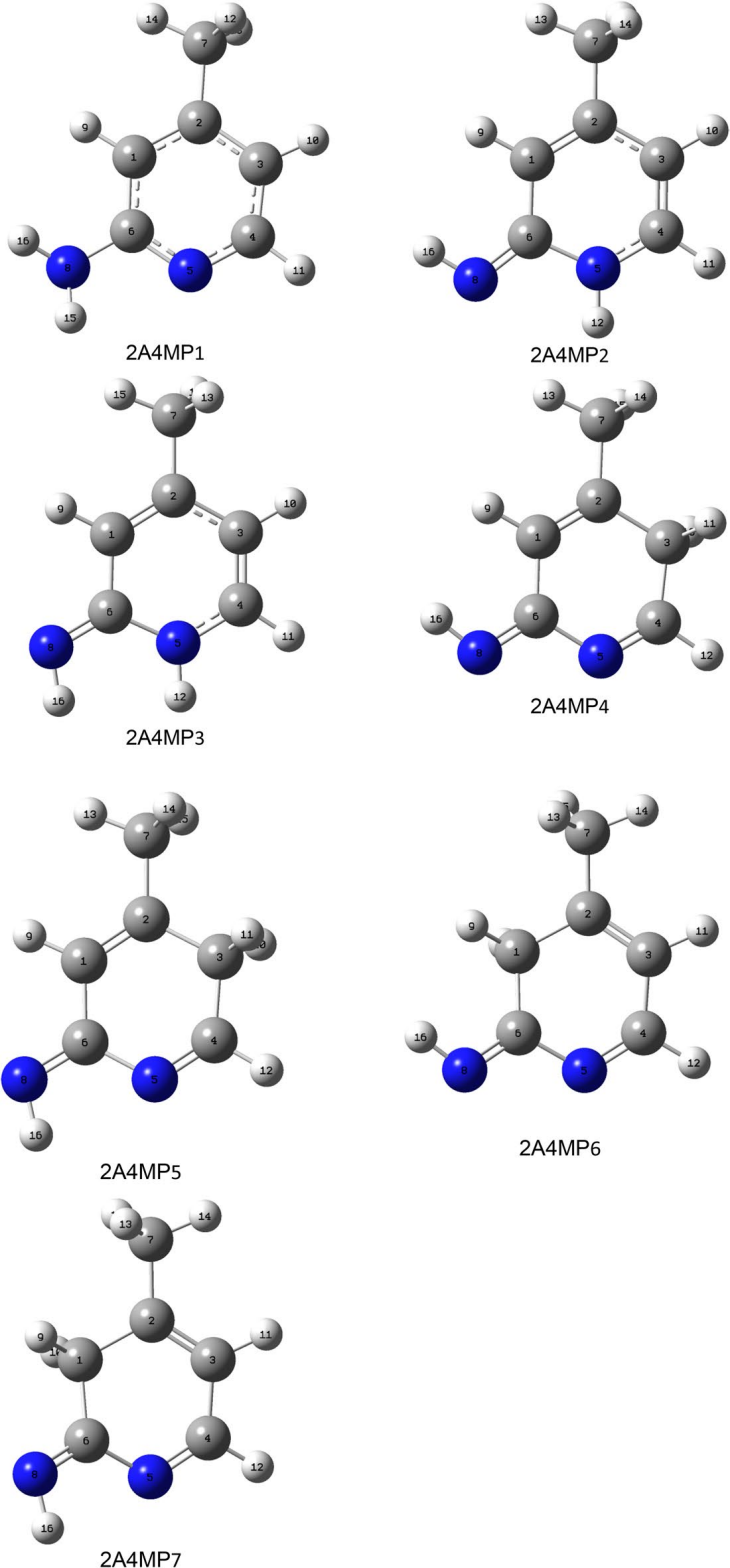
Optimized structures of common and rare tautomers of 2A4MP

Tautomerism is crucial for the understanding of the chemical behavior of 2A4MP. All possible tautomers of 2A4MP were optimized at B3LYP/6-311++G(d,p) and shown in Fig. 2. Relative energy (kcal/mol) for 2A4MP tautomers at B3LYP/6-311++G(d,p) computational levels is presented in Table 1. The canonical structure of the 2A4MP1 is the most stable tautomer. It is 13.60 kcal/mole more stable than 2A4MP2. Comparing the cis (2A4MP3) and trans (2A4MP2) of 2(1H)-pyridinimine reveals that, the trans tautomer is 2.76 kcal/mol more stable than the cis one. This result agrees with the reported experimental and computational (at B3LYP/6-31++G(d,p) level) data of 2-aminopyridine/2(1H)-pyridinimine (Akai and Ohno 2005). The destabilization of the cis tautomer may result from the interaction of the neighboring H atoms. As could be predicted from the computed total energy values which are, corrected for zero-point energy, one may rank the stability order as 2A4MP1 > 2A4MP2 > 2A4MP3 > 2A4MP5 > 2A4MP7 > 2A4MP4 > 2A4MP6. The attachment of the movable H atom to N (2A4MP2 and 2A4MP3) is more favored than to C (2A4MP4, 2A4MP5, 2A4MP6, and 2A4MP7). Bonding of the movable H to C3 (2A4MP5 and 2A4MP7) is also more stable than bonding to C5 (2A4MP4 AND 2A4MP6) by about 4 kcal/mol.

**Table 1:** Relative energy (kcal/mol) for 2A4MP tautomers at B3LYP/6-311++G(d,p) computational levels

2A4MP1	2A4MP2	2A4MP3	2A4MP4	2A4MP5	2A4MP6	2A4MP7
0	13.60	16.35	32.05	27.96	33.55	28.69

### Transition state for hydrogen transfer

**Fig. 3:**
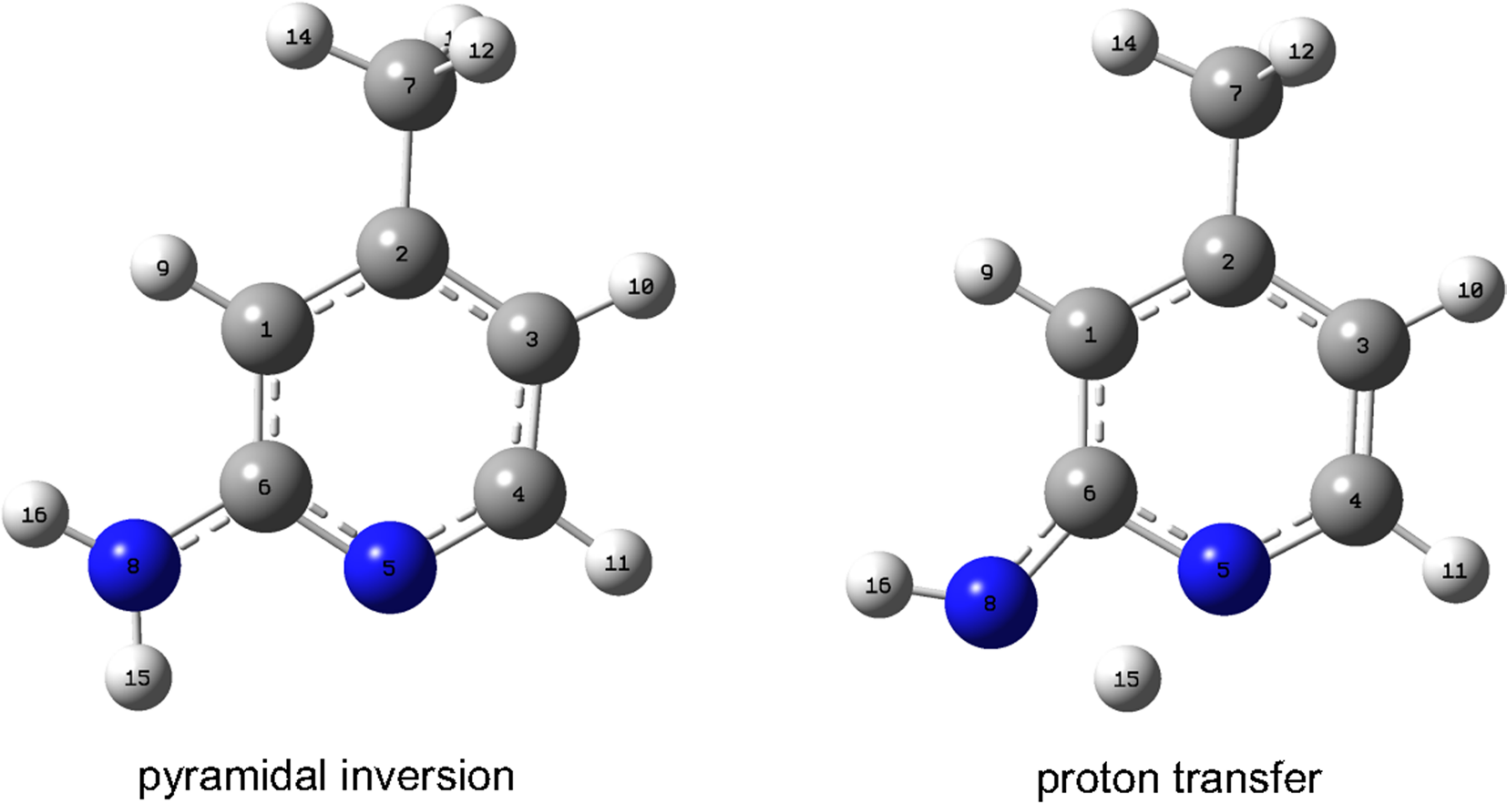
Optimized transition state structures for pyramidal inversion and proton transfer

The transition state structure for proton transfer within the amine-imine tautomerism was calculated at B3LYP/6-311++G(d,p) and confirmed by the presence of one imaginary frequency. The transition state structure for proton transfer is shown in Fig. 3. The imaginary frequency value for hydrogen transfer is (−1897 cm^−1^). The curvature of the potential energy surface (PES) for the proton transfer reaction is measured by the second derivative of energy with respect to geometry. The magnitude of the imaginary frequency is a measure of the curvature of the transition state region along the reaction coordinate. For the proton transfer related reactions, a small imaginary frequency should correspond to a low barrier and a large imaginary frequency to a high barrier. The higher values of the imaginary frequency of the proton transfer reactions are because the vibrational frequencies are mass-weighted eigenvalues of the force constant matrix (second derivative matrix). Therefore, light atoms e.g. the H atom, is associated with higher vibrational frequency transition state. The activation energy of the proton transfer process is computed as 44.81 kcal/mol at B3LYP/6-311++G(d,p) level of theory. At the DFT/B3LYP/6-311++G** level, the calculated energy barrier for 2-hydroxypyridine → 2-pyridone conversion is about 34.00 kcal/mol (Hazra and Chakraborty 2006). It is believed that examination of key molecular orbitals (like HOMO) may provide insight into molecular geometry. The HOMO energy of the transition state is calculated to be -135.76 kcal/mol. The difference in HOMO energy between the transition state and the ground state is found to be 3.53 kcal/mol. The low-lying HOMO is interacting with the moving proton in such a way that facilitate the transfer of the proton and hence the lower the activation barrier. The geometry of transition state structures are given in Additional file 1: Table S5. The transferred proton shows a longer bond distance (1.395 Å) compared to that of the minimum structure bond distance (1.009 Å). The amino group in the transition state is planar as evidenced from the dihedral angles D(1,6,8,15), D(1,6,8,16), D(5,6,8,15) and D(5,6,8,16) as shown in Table S5.

### Pyramidal inversion barrier at amino-N

The inversion of pyramidal centers is an important process, and takes place very rapidly in the case of amines. The inversion barrier at the N atom is calculated as the difference of total energy between the planar transition state structure (saddle point) and the unconstrained optimized pyramidal structure (local minimum). The transition state structure of pyramidal inversion is displayed in Fig. 3. The activation energy of pyramidal inversion is calculated to be 0.41 kcal/mol at B3LYP/6-33++G(d,p). The rapid rate of inter-conversion in the case of 2-aminopyridine with activation energy of 0.41 kcal/mol is much less than in the case of other pyramidal compounds of elements from the second row and higher (Montgomery 2013). The imaginary frequency of pyramidal inversion at the transition state structure is computed as −346 cm^−1^. The experimental vibration is reported as 289–375 cm^−1^ in the gas phase (McKean 1989). The computed inversion to barrier is 0.41 kcal/mol which is less than the computed value of 0.88 kcal/mol at HF/6-31G(d) (Bludský et al. 1996) and the estimated value near 0.70 kcal/mol for 2-aminopyridine (Barrow et al. 1976).

The HOMO energy of the pyramidal optimized structure and the planar transition state are calculated as −139.29 and −126.32 kcal/mol respectively. The difference of HOMO energies is 12.98 kcal/mol. The smaller the difference the more rapid the inversion. The HOMO contains the lone electron pair and one would expect that this orbital should be an *sp3* orbital in the pyramidal structure and a *p* orbital in the planar case. The HOMO energy difference in NMe_3_ is reported 23.81 kcal/mol, and the inversion barrier is 8.38 kcal/mol (Montgomery 2013). It is found that, the HOMO energy increases moving from the pyramidal ground state to the planar transition state.

### Natural charges and bond order of 2A4MP1

One of the most important electronic properties is the population of electronic density on atoms constituting the molecule. A good way to explore the distribution of charge within a system is to calculate the natural charge. The values of the NPA charges for all atoms in 2A4MP1 were computed at B3LYP/6-311+G(d,p). NPA charges for 2A4MP1 are given in Additional file 1: Table S6. In Gaussian, NBO analysis calculates atomic charges, by summing occupancy of natural atomic orbitals. In 2A4MP positive charges are accommodated on some carbon atoms (C2, C4 and C6) and H atoms. These carbon atoms (C4 and C6) are either directly bonded to the more electronegative N atoms or in the meta position (C2) with regard to the amino group. N atoms and some carbon atoms (C1 and C7) accommodate negative charge. N8 is highest negatively charged atom (−0.792).

Bond order of 2A4MP1 molecule is computed at the level B3LYP/6-311+G(d,p). The spin-corrected Mayer bond order in the natural atomic basis (NAO), is shown in Additional file 1: Table S7. Since 2A4MP1 is spin unpolarized system, the spin-corrected Mayer bond order in the NAO basis equals the “Wiberg bond index in the NAO basis” as implemented in Gaussian NBO Version 3.1. As can be seen from Additional file 1: Table S7, the highest calculated bond order is for the bond C3–C4 (1.463) and the smallest computed bond order for bonded atom pairs is N8-H15 (0.831). The bond order between C1–C2 is 1.458 indicating a partial double bond and that between C1–C7 is 1.036 indicating a pure single bond. The bond order C6–N5 is 1.369 which suggests a partial double bond while the bond order of the C6–N8 bond is 1.145 showing some partial double bond character which results from N lone pair delocalization.

### Vibrational assignments

**Fig. 4:**
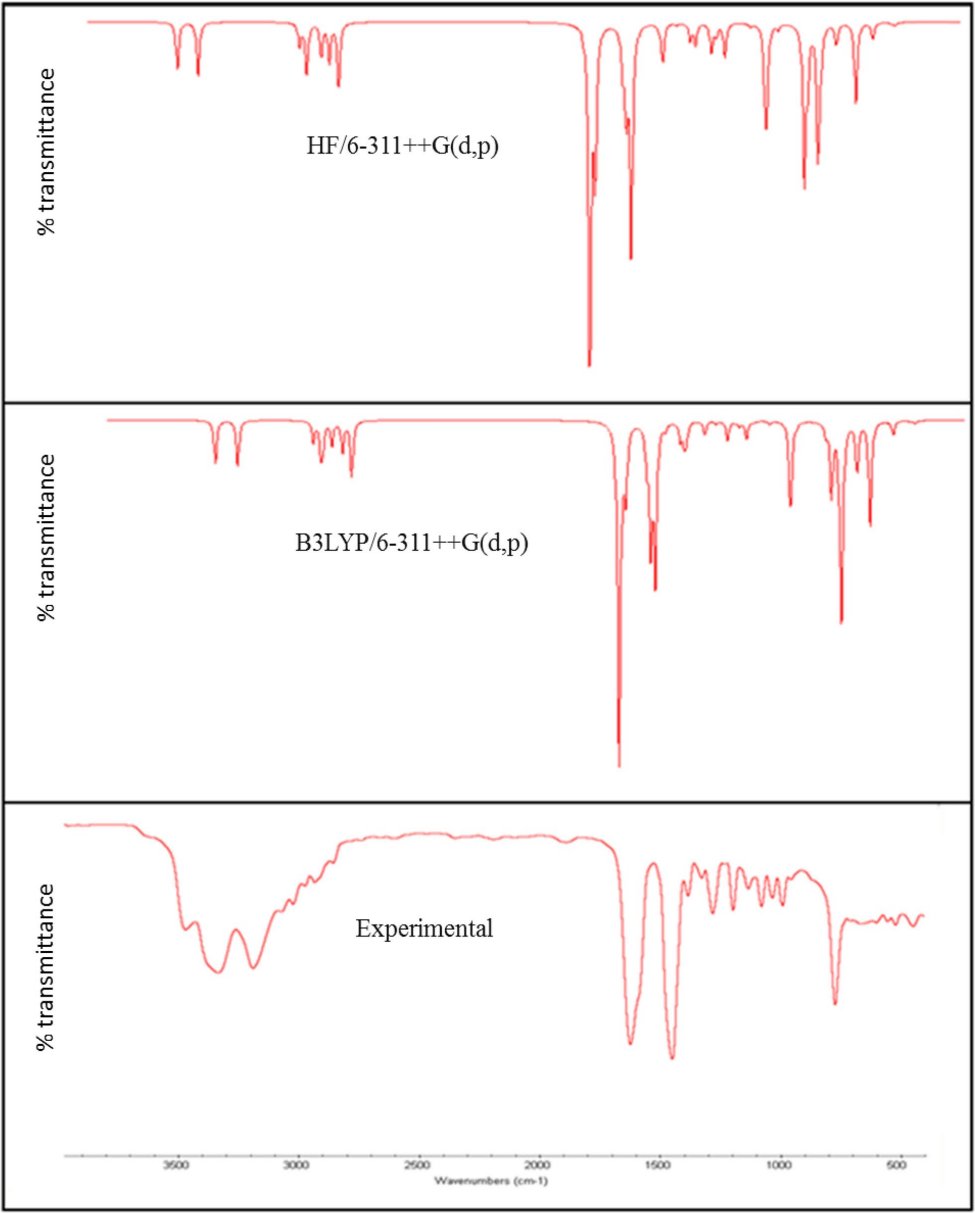
Comparison of experimental and calculated IR spectra of 2-amino-3-methylpyridine

**Fig. 5:**
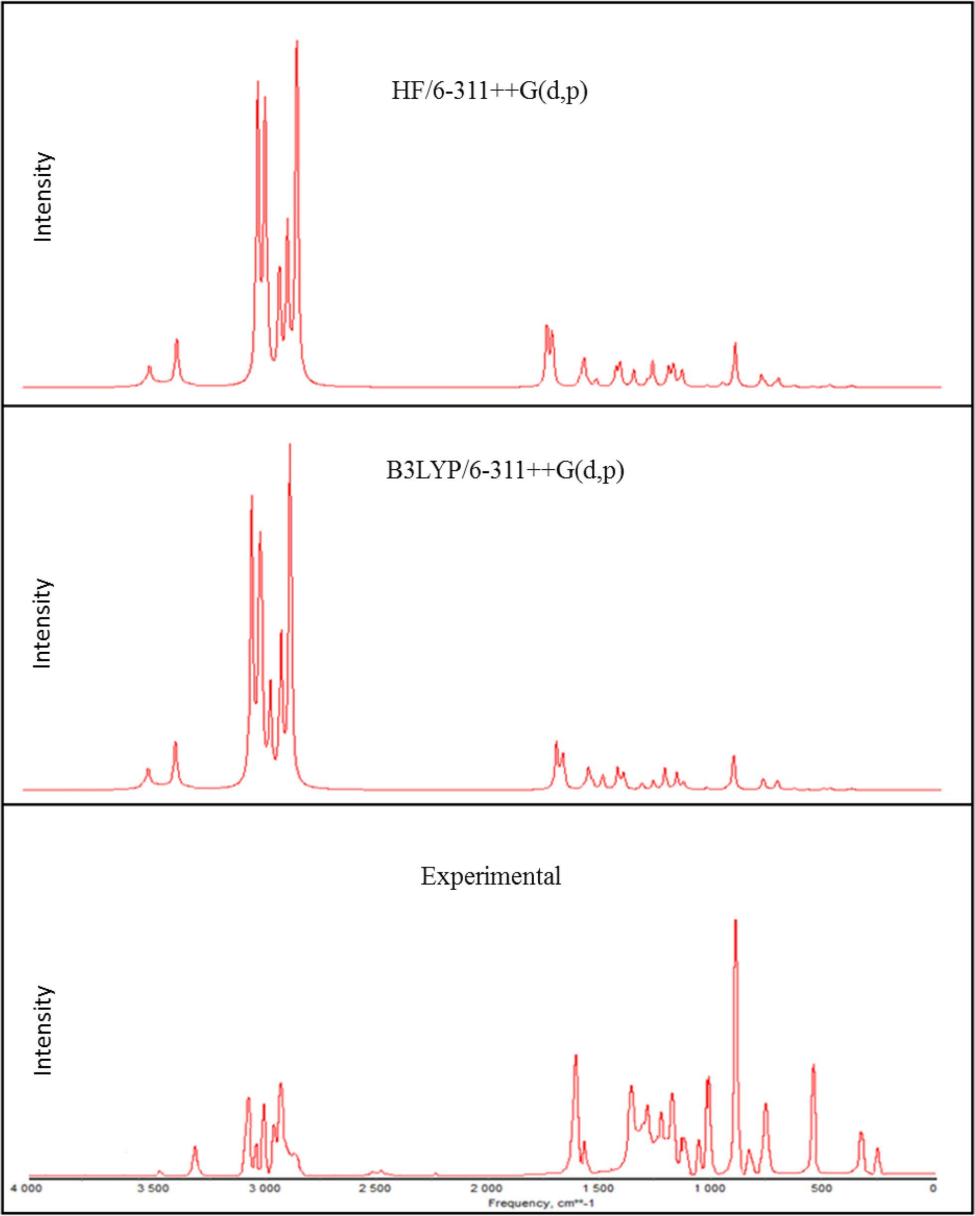
Comparison of experimental and calculated Raman spectra of 2-amino-3-methylpyridine

**Fig. 6:**
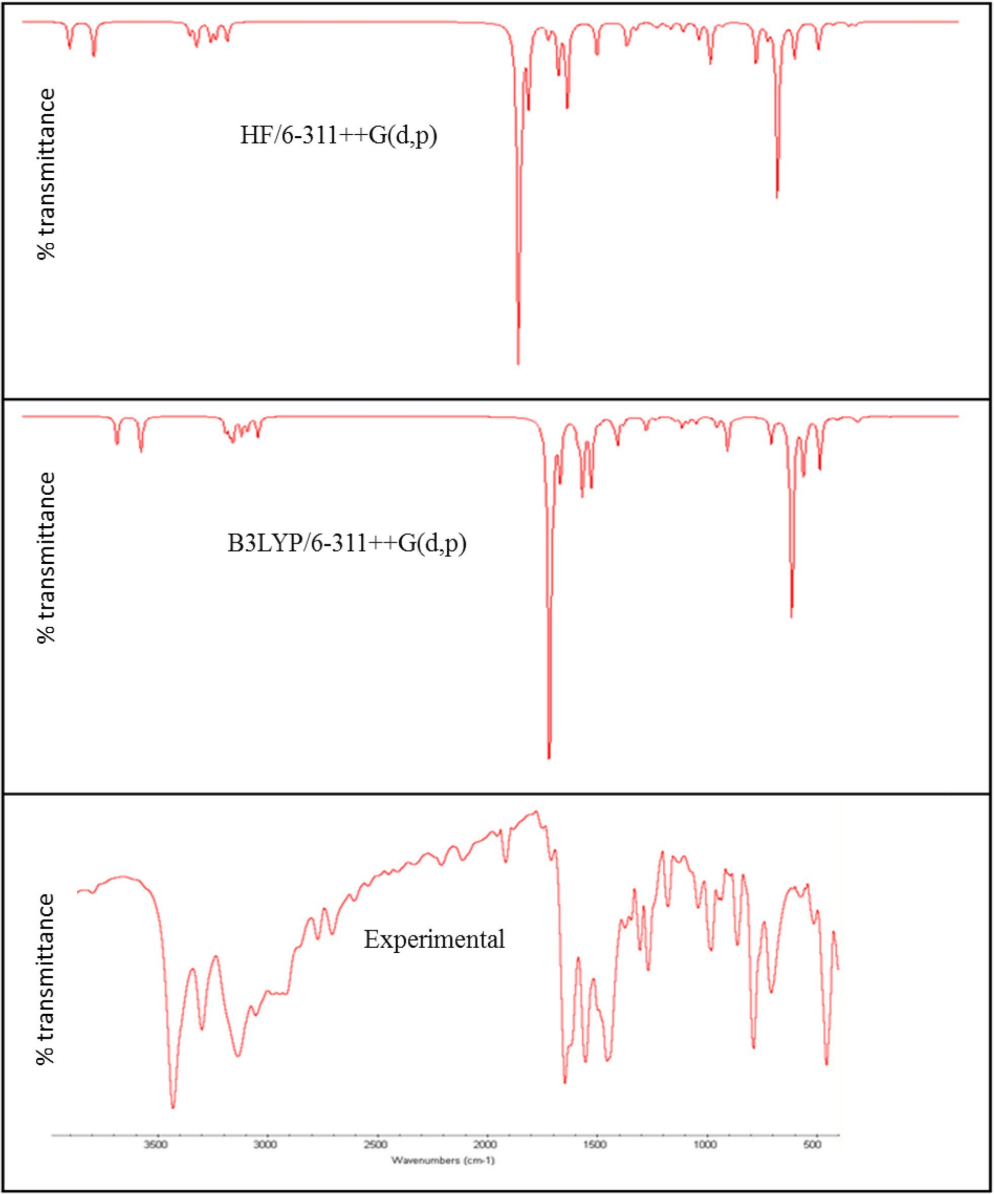
Comparison of experimental and calculated IR spectra of 2-amino-4-methylpyridine

**Fig. 7:**
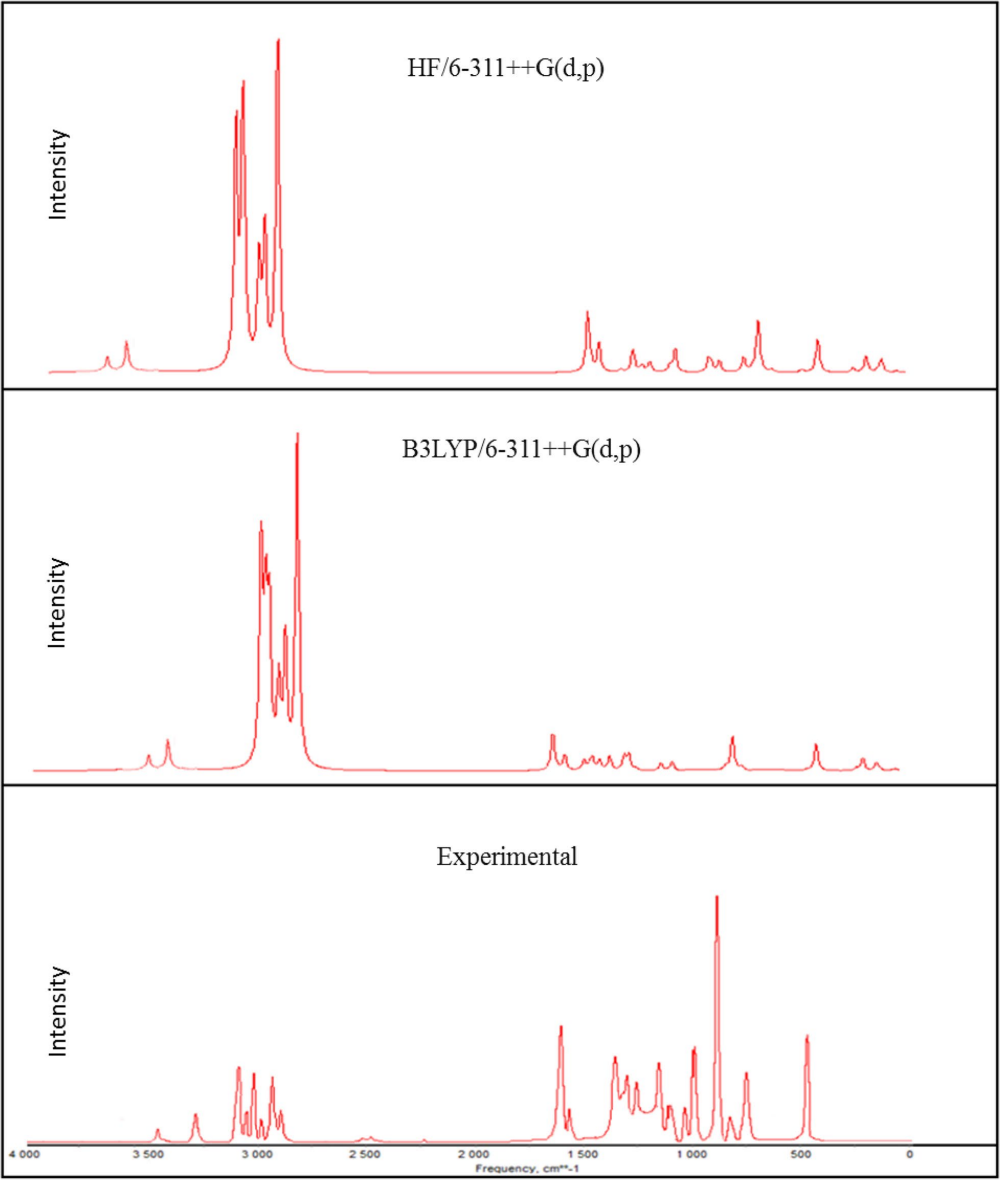
Comparison of experimental and calculated Raman spectra of 2-amino-4-methylpyridine

2A3MP and 2A4MP molecules belong to C_1_ point group symmetry, having 16 atoms with 42 normal modes of vibrations which are active in both Raman scattering and IR absorption. The vibrational analysis of fundamental modes with FT-IR and FT- Raman experimental frequencies are tabulated in Table 2 for 2A3MP and 2A4MP. Vibrational frequencies using HF, B3LYP and MP2 methods with different basis sets, for 2A3MP and 2A4MP were reported in Tables 3 and 4 respectively. The experimental Infrared and Raman spectra in solid phase were shown in Figs. 4 and 5 for 2A3MP and Figs. 6 and 7 for 2A4MP along with calculated IR and Raman spectra by HF and B3LYP methods with 6-31++G(d,p) and 6-311++G(d,p) basis sets.

**Table 2:** Experimental FT-IR, FT-Raman frequencies and assignment for 2A3MP and 2A4MP

FT-IR frequency (cm^−1^)		FT-Raman frequency (cm^−1^)		Assignment
2A3MP	2A4MP	2A3MP	2A4MP	
3470	3431	3468	3430	NH_2_ asym. stretch
3335	3300	3338	3305	NH_2_ sym. stretch
3068	3096	3067	3095	CH asym. stretch
–	–	3033	3060	CH stretch
3024	3055	3019	3041	CH stretch
2974	2980	2980	2980	CH_3_ asym. stretch
2935	2919	2925	2955	CH_3_ asym. stretch
2858	–	2864	2900	CH_3_ sym. stretch
1623	1646	1618	1646	NH_2_ scissoring
1449	1453	–	–	CH_3_ asym. scissoring
1384	1374	1379	1370	CH_3_ sym. scissoring
1282	1268	1264	1270	C=N stretch
1250	1248	1195	1246	CH in- plane bending
1196	1177	1188	1177	C–CH_3_ stretch
1135	1130	1137	1130	CH in- plane-bending
1080	1040	1068	1038	CH in- plane-bending
1035	1035	1031	1033	CH_3_ rocking
–	–	855	830	CH out-of-plane
774	788	762	786	CH out-of-plane
–	–	750	750	CH out-of-plane
524	455	525	455	NH_2_ wagging
–		288		C–CH_3_ in-plane-bending
–		251		C–CH_3_ out-of-plane

**Table 3:** Calculated fundamental frequencies of 2A3MP at different levels

Mode of vibration	Calculated frequency (cm^−1^)												Vibrational assignments
	HF/6-311++G(d,p)				B3LYP/6-311++G(d,p)				MP2/6-31G(d)		MP2/6-31G++(d,p)		
	Wave number		IR intensity	Raman intensity	Wave number		IR intensity	Raman intensity	Wave number	IR intensity	Wave number	IR intensity	
	Unscaled	Scaled			Unscaled	Scaled			Unscaled		Unscaled		
1	3914	3483	33.46	38.59	3620	3475	33.46	38.59	3681	22.33	3739	28.00	NH_2_ asym. stretch
2	3803	3385	38.28	128.04	3478	3339	38.28	128.04	3562	30.68	3612	29.03	NH_2_ sym. stretch
3	3359	2990	17.13	158.09	3195	3067	17.13	158.09	3255	13.85	3280	16.47	CH stretch
4	3323	2957	30.04	71.59	3158	3032	30.04	71.59	3218	6.42	3248	1.12	CH stretch
5	3317	2952	8.31	89.51	3148	3022	8.31	89.51	3213	22.11	3240	23.15	CH stretch
6	3243	2886	23.06	57.57	3101	2977	23.06	57.57	3199	10.90	3215	12.60	CH_3_ asym. stretch
7	3200	2848	28.38	81	3048	2926	28.38	81.00	3163	12.23	3177	12.32	CH_3_ asym. stretch
8	3151	2804	45.23	179.66	2977	2857	45.23	179.66	3087	16.87	3098	23.71	CH_3_ sym. stretch
9	1804	1606	23.06	31.04	1692	1624	23.06	31.04	1697	224.58	1680	232.70	NH_2_ scissoring + C=C stretch
10	1778	1582	37.92	12.05	1627	1562	37.92	12.05	1666	27.45	1655	42.39	C=C stretch +NH_2_ scissoring
11	1775	1580	64.76	16	1615	1550	64.76	16.00	1661	24.71	1648	27.98	C–C stretch
12	1630	1451	28.89	3.63	1509	1449	28.89	3.63	1564	55.80	1547	44.84	CH_3_ asym. scissoring
13	1611	1434	53.25	7.83	1489	1429	53.25	7.83	1542	2.25	1514	18.42	C=C stretch
14	1604	1428	7.64	8.99	1485	1426	7.64	8.99	1521	7.14	1510	8.95	CH_3_ sym. scissoring
15	1583	1409	162.33	2.14	1466	1407	162.33	2.14	1512	76.96	1497	79.28	C=C stretch + CH_3_ sym. scissoring
16	1540	1371	1.55	4.03	1439	1381	1.55	4.03	1466	2.02	1451	1.16	CH_3_ sym. scissoring
17	1436	1278	3.3	9.46	1339	1285	3.30	9.46	1439	22.30	1436	29.75	C-NH_2_ stretch + CC stretch
18	1414	1258	27.74	12.33	1320	1267	27.74	12.33	1356	1.27	1345	1.36	C = N stretch
19	1341	1193	1.46	9.08	1309	1257	1.46	9.08	1342	8.57	1332	12.77	CH in-plane-bending
20	1267	1128	12.3	2.94	1245	1195	12.30	2.94	1258	7.97	1251	11.77	C–CH3 stretch
21	1240	1104	16.16	13.77	1185	1137	16.16	13.77	1195	0.94	1181	1.02	CH in-plane-bending
22	1162	1034	0.15	0.13	11,024	1079	0.15	0.13	1132	0.88	1122	0.91	CH in-plane-bending
23	1155	1028	20.89	9.88	1077	1033	20.89	9.88	1077	3.27	1064	3.24	CH_3_ rocking
24	1129	1005	8.88	11.25	1041	999	8.88	11.25	1075	5.68	1055	4.63	Ring breathing
25	1067	950	0.55	0.91	1006	966	0.55	0.91	1032	3.53	1025	2.90	Trigonal bending
26	1083	964	24.24	8.69	972	933	1.223	0.41	919	7.60	896	9.54	CH out-of-plane
27	1067	950	0.98	0.12	950	912	0.296	0.04	900	3.24	894	3.89	CCC in-plane-bending
28	946	842	2.74	0.99	888	852	2.74	0.99	893	0.79	862	0.16	CH out- of- plane
29	868	773	46.55	1.53	786	755	46.55	1.53	790	45.68	767	10.41	CH out- of- plane
30	860	765	47.18	0.97	779	748	47.18	0.97	770	9.62	764	35.11	CH out- of- plane
31	799	711	4.09	23.58	754	724	4.09	23.58	744	266.64	712	276.48	CCC in-plane-bending
32	658	586	110.56	6.15	606	582	110.56	6.15	648	109.60	601	59.48	CH_2_ + twisting in CH_3_
33	641	570	31.27	2.13	577	554	31.27	2.13	602	6.33	568	13.57	CNC in-plane-bending
34	586	522	97.34	1.76	560	537	97.34	1.76	539	22.44	535	2.78	CCC in-plane-bending
35	567	505	17.59	4.59	542	520	17.59	4.59	532	4.29	487	15.88	NH_2_ wagging
36	489	435	13.89	0.58	449	431	13.89	0.58	447	19.35	445	1.42	C–NH_2_ in-plane bending
37	479	426	1.31	0.48	444	426	1.31	0.48	424	1.26	378	15.45	CCC out-of-plane + CNC out-of-plane
38	383	341	56.96	0.58	382	367	56.96	0.58	371	37.72	331	30.52	C–NH_2_ out-of-plane
39	325	289	0.24	0.48	300	288	0.24	0.48	301	2.93	301	2.69	C–CH_3_ in-plane bending + C-NH_2_ in-plane bending
40	294	262	11.34	1.41	266	255	11.34	1.41	269	3.93	232	3.63	C–CH_3_ out-of-plane + C-NH_2_ in-plane bending
41	195	174	0.81	0.16	178	171	0.81	0.16	184	3.74	177	3.69	CCC out-of-plane
42	174	155	2.52	0.91	156	150	2.52	0.91	158	0.34	146	0.24	CH_3_ torsion

**Table 4:** Calculated fundamental frequencies of 2A4MP at different levels

Mode of vibration	Calculated frequency (cm^−1^)												Vibrational assignments
	HF/6-311++G(d,p)				B3LYP/6-311 ++G(d,p)				MP2/6-31G(d)		MP2/6-31G++(d,p)		
	Wave number		IR intensity	Raman intensity	Wave number		IR intensity	Raman intensity	Wave number Unscaled	IR intensity	Wave number Unscaled	IR intensity	
	Unscaled	Scaled			Unscaled	Scaled							
1	3915	3484	35.10	43.27	3572	3429	29.12	54.97	3692	22.36	3748	28.00	NH_2_ asym. stretch
2	3803	3385	45.10	137.44	3449	3311	38.09	206.13	3572	30.72	3619	29.03	NH_2_ sym. stretch
3	3350	2982	16.54	123.71	3266	3135	14.04	149.71	3239	13.72	3265	16.47	CH stretch
4	3321	2956	9.81	90.05	3192	3064	14.35	99.14	3217	6.33	3242	1.12	CH stretch
5	3315	2950	24.65	65.81	3179	3051	22.20	98.07	3212	22.09	3240	23.15	CH stretch
6	3251	2893	24.22	55.49	3106	2982	18.00	55.85	3197	10.91	3216	12.60	CH_3_ asym. stretch
7	3227	2872	19.50	69.72	3078	2955	13.02	86.15	3182	12.32	3198	12.32	CH_3_ asym. stretch
8	3173	2824	25.08	173.18	3027	2906	21.58	228.80	3101	16.92	3112	23.71	CH_3_ sym. stretch
9	1800	1602	41.26	30.49	1712	1643	35.17	25.38	1703	224.65	1690	232.70	NH_2_ scissoring + C=C stretch
10	1786	1590	72.47	3.54	1635	1570	34.32	1.70	1684	27.56	1669	42.39	C=C stretch +NH_2_ scissoring
11	1750	1558	51.56	15.42	1601	1537	57.64	10.37	1646	24.71	1636	27.98	C–C stretch
12	1656	1474	19.35	1.45	1590	1526	17.05	7.38	1558	55.80	1539	44.84	CH_3_ asym. scissoring
13	1609	1432	1.95	3.37	1556	1493	76.44	2.69	1548	2.25	1532	18.42	C=C stretch
14	1605	1428	0.25	8.79	1512	1451	7.62	8.81	1545	7.14	1522	8.95	CH_3_ sym. scissoring
15	1568	1396	43.00	3.71	1454	1396	2.83	6.68	1501	76.96	1486	79.28	C=C stretch + CH_3_ sym. scissoring
16	1535	1366	1.95	5.46	1412	1356	3.62	9.65	1467	2.02	1451	1.16	CH_3_ sym. scissoring
17	1451	1291	0.25	3.07	1351	1297	5.84	9.76	1426	22.30	1420	29.75	C–NH_2_ stretch + CC stretch
18	1429	1272	43.00	11.87	1331	1278	29.93	10.73	1368	1.27	1357	1.36	C=N stretch
19	1288	1146	24.37	5.99	1302	1250	6.23	1.55	1351	8.57	1340	12.77	CH in-plane-bending
20	1277	1137	16.11	4.36	1224	1175	14.35	4.78	1232	7.97	1222	11.87	C–CH3 stretch
21	1243	1106	8.55	5.81	1172	1125	2.56	5.91	1185	0.94	1172	1.02	CH in-plane-bending
22	1156	1029	4.78	0.06	1081	1037	0.62	0.53	1118	0.88	1106	0.91	CH in-plane-bending
23	1139	1014	5.70	7.65	1073	1030	3.77	0.06	1081	3.27	1066	3.24	CH_3_ rocking
24	1105	983	0.04	0.74	1002	958	11.18	2.83	1052	5.68	1045	4.63	Ring breathing
25	1099	978	3.54	1.09	998	958	5.90	23.56	1019	3.53	1017	2.90	Trigonal bending
26	1078	959	7.91	26.89	978	939	0.43	0.26	976	7.60	968	9.54	CH out-of-plane
27	1020	908	12.26	1.48	959	921	6.84	2.83	922	3.24	902	3.29	CCC in-plane-bending
28	947	843	22.00	0.20	863	828	10.45	0.08	835	0.59	811	0.16	CH out-of-plane
29	893	795	54.97	1.40	813	780	36.36	0.69	793	45.98	783	10.41	CH out-of-plane
30	836	744	4.24	1.04	777	746	1.00	18.62	787	9.82	771	35.11	CH out-of-plane
31	827	736	0.18	16.53	759	729	1.16	0.28	728	266.44	699	276.48	CCC in-plane-bending
32	678	603	53.40	2.24	607	583	26.71	1.72	659	109.50	608	59.48	CH_2_ + twisting in CH_3_
33	623	554	16.96	8.29	759	729	2.28	8.49	579	6.33	577	13.57	CNC in-plane-bending
34	576	513	226.60	0.96	523	502	4.10	5.15	545	22.34	523	2.88	CCC in-plane-bending
35	559	498	2.48	6.74	477	457	207.64	1.28	525	4.39	476	15.88	NH_2_ wagging
36	496	441	44.98	0.85	454	436	55.99	0.58	443	19.25	434	1.42	C–NH_2_ in-plane bending
37	469	417	2.05	0.20	439	421	6.02	1.19	436	1.26	414	15.25	CCC out-of-plane + CNC out-of-plane
38	383	341	36.68	0.60	377	362	55.61	0.50	345	37.72	343	30.42	C–NH_2_ out-of-plane
39	313	279	2.93	0.76	294	282	2.76	0.78	294	2.89	292	2.69	C–CH_3_ in-plane bending + C-NH_2_ in-plane bending
40	241	214	4.88	0.99	210	202	2.08	0.86	211	3.83	200	3.53	C–CH_3_ out-of-plane + C-NH_2_ in-plane bending
41	210	187	5.25	0.73	197	189	5.24	0.91	196	3.64	185	3.59	CCC out-of-plane
42	71	63	0.09	0.25	147	141	0.31	0.37	36	0.17	83	0.14	CH_3_ torsion

### C–H vibrations

Aromatic C–H stretching vibrations normally occur at 3100–3000 cm^−1^ (Sharma 1994). The 2A3MP and 2A4MP are di-substituted aromatic systems. The expected three C–H symmetric stretching vibrations correspond to mode nos. 3, 4 and 5 in Table 2.

The calculated frequencies of the C-H symmetric stretching vibrations using B3LYP/6-311++G(d,p) in 2A3MP at 3067, 3032 and 3022 cm^−1^ are in good agreement with the experimental data. Similar observations are also found in the case of 2A4MP at 3135, 3064 and 3051 cm^−1^. The C–H in-plane bending and C–H out-of-plane bending vibrations are normally found in the range 1300–1000 and 1000–750 cm^−1^, respectively in aromatic compounds and are very useful for characterization purposes (Prabavathi and Krishnakumar 2009; Krishnakumar and Prabavathi 2008; Altun et al. 2003; Singh and Pandey 1974).

In the case of 2A3MP three modes are associated mainly with the C–H in-plane-bending vibrations: mode nos. 19, 21 and 22. These modes are at the following wavenumbers 1257, 1137 and 1079 cm^−1^ and at 1250, 1125 and 1037 cm^−1^ in the case of 2A4MP. The C–H out-of-plane bending vibrations correspond mainly to the mode nos. 28, 29 and 30. The calculated frequencies of the C–H out-of-plane bending vibrations in 2A3MP at 852, 755 and 748 cm^−1^ and in 2A4MP at 828, 780 and 746 cm^−1^ show very good agreement with experimental data.

### NH_2_ vibrations

There are six internal modes of vibrations in the NH_2_ group. These are the symmetric stretching, the anti-symmetric stretching, the scissoring, the rocking, the wagging and torsional mode. The NH_2_ group has two (N–H) stretching vibrations, one being asymmetric and another symmetric. The –NH_2_ symmetric and asymmetric stretches in the range (3311–3475 cm^−1^) (mode 1) and (mode 2) are in good agreement with experimental value of (3470–3300 cm^−1^) in both 2A3MP and 2A4MP molecules. The computed –NH_2_ scissoring vibration mode no. (9) at 1624 and 1643 cm^−1^ for 2A3MP and 2A4MP, respectively by B3LYP/6-311++G(d,p) level are in excellent agreement with the expected characteristic value, 1600 cm^−1^ (Yadav and Sing 1985; Wiberg and Shrake 1973). This band observed in FT-IR at 1623 cm^−1^ and in FT-Raman at 1618 cm^−1^ for 2A3MP and at 1646 cm^−1^ in both FT-IR and FT-Raman spectra for 2A4MP. The –NH_2_ wagging computed at 520 and 457 cm^−1^ (mode 35) in both 2A3MP and 2A4MP molecules using B3LYP/6-311++G(d,p) level have been observed at 524 and 525 cm^−1^ in FT-IR and FT-Raman spectra, respectively for 2A3MP and in the case of 2A4MP observed at 455 cm^−1^ in both FT-IR and FT-Raman spectra.

### Methyl group vibrations

Nine modes of vibration (Sundaraganesan and Doinic 2007) are assigned to each CH_3_ group; the symmetrical and asymmetrical stretching, in plane stretching modes, deformation modes (symmetrical and asymmetrical), in-plane and out-of-plane rocking, and twisting modes. Stretching frequency of methyl C–H usually occurs at lower frequencies (2800–3000 cm^−1^) than those of the aromatic ring normally occurs at 3000–3100 cm^−1^. Stretching vibrations of methyl C-H, ν_sym_ and ν_asym_, generally found in the ranges 2850–2940 and 2970–3010 cm^−1^ (Sathyanarayana 2004; Lithivinov 1992; Furic et al. 1992). The reported spectra of 2-methyl pyridine, shows the CH_3_ stretching in the range 2900–3000 cm^−1^, the in-plane deformations around 1370–1450 cm^−1^ and the rocking around 990–1040 cm^−1^ (Furic et al. 1992). The measured wavenumbers of asymmetric and symmetric stretching vibrations of CH_3_ group for both molecules are listed in Table 2. For the methyl substituted benzene derivatives the symmetric and asymmetric deformation vibrations ν_def_ of methyl groups normally appear in the region 1370–1390 and 1440–1465 cm^−1^, respectively (Arenas et al. 1997; Lin-Vien et al. 1991; Diem 1993). The rocking mode of vibrations of the CH_3_ group usually appear in the region 1010–1070 cm^−1^ (Silverstein et al. 1981). In the present study, this band appears at 1035 cm^−1^ in FT-IR and at 1031 cm^−1^ in FT-Raman (mode no. 23) for 2A3MP. The same vibration in 2A4MP is shown in FT-IR at 1035 cm^−1^ and at 1033 cm^−1^ in FT-Raman. The theoretically calculated values by MP2 and B3LYP method agree well with the experimental values. CH_3_ torsional mode is expected below 400 cm^−1^, the computed bands at 150 cm^−1^ in 2A3MP and 141 cm^−1^ in 2A4MP are assigned to this mode (mode no. 42).

### C–CH3 vibrations

In 2A3MP, the band observed in FT-IR at 1196 cm^−1^ and at 1188 cm^−1^ in FT -Raman is assigned to C–CH3 stretching vibration. It is found that there is a good agreement between this experimental value and the calculated value mode no. 20 at 1195 cm^−1^ using B3LYP/6-311++G(d,p) method. A similar band is predicted at 1175 cm^−1^ for 2A4MP shows good agreement with the experimental value at 1177 cm^−1^ in FT-IR and FT-Raman. In methyl substituted benzenes, the C-CH3 in-plane-bending vibrations give rise to absorptions in the range 390–260 cm^−1^. The C–CH_3_ in-plane bending vibration mode no. 39 is assigned at 288 and 282 cm^−1^ for 2A3MP and 2A4MP, respectively and out-of-plane bending vibration mode No. 40 is assigned at 255 cm^−1^ for 2A3MP and 202 cm^−1^ for 2A4MP. These assignments are in good agreement with the literature (Hameka and Jensen 1996).

### C–N, C=N vibrations

The identification of C–N vibrations is very difficult because of the interference of many bands in the area where the vibration of this bond happens. For the aromatic amines, the C-N stretching appears in the region 1266–1382 cm^−1^ (Silverstein et al. 1981). This band observed, in 3,5-dibromopyridine, at 1410 cm^−1^ in FT-IR and 1412 cm^−1^ in FT-Raman. The band observed at 1368 cm^−1^ in benzamide is assigned to C–N stretching (Shanmugam and Sathyanarayana 1984). In the current study, a band appeared at 1282 and 1264 cm^−1^ for the stretching vibration of the C=N bond in the FT-IR and FT-Raman, respectively for 2A3MP and at 1268 and 1270 cm^−1^ for the stretching vibration of the C=N bond in the FT-IR and FT-Raman, respectively for 2A4MP. These values are consistent with the theoretically calculated values (mode No. 18).

## Conclusion

In this work, the structure and vibrational analysis of 2A3MP and 2A4MP were investigated by MP2/6-31G(d) and MP2/6-31++G(d,p) methods. Tautomerization of 2A4MP was studied by DFT/B3LYP method in the gas phase. In this study, all possible tautomers including NH → NH conversions as well as, NH → CH and CH → CH, were considered. The most stable tautomer is the canonical structure, 2A4MP1. The umbrella effect transition state structure of the pyramidal N and transition structure of the proton transfer were computed at B3LYP/6-311++G(d,p). Activation energy of the hydrogen proton transfer and pyramidal inversion at amino N is calculated as 44.81 and 0.41 kcal/mol respectively. Bond order and natural atomic charges were also calculated.

The vibrational spectral analysis was carried out using FT-IR and FT- Raman spectroscopy for 2A3MP and 2A4MP. The computations were performed at ab initio (MP2) and DFT (B3LYP) levels of theory with 6-31++G(d,p) and 6-311++G(d,p) basis sets to get the optimized geometry and vibrational wavenumbers of the normal modes of the title compounds. The complete vibrational assignments of wavenumbers were made on the basis of potential energy distribution and using Gauss View software. The difference between the observed and scaled wave number values of the most of the fundamentals is very small.

### Experimental details

The compounds 2A3MP and 2A4MP were purchased from Sigma–Aldrich with a stated purity of 99 % and were used without further purification. FT-IR spectra of 2A3MP and 2A4MP have been recorded in the region 4000–400 cm^−1^ using a Thermo Nicolet Nexus 870 FT-IR instrument. The instrument is equipped with a KBr beam splitter and an In GaAs detector. The spectral resolution is ±2 cm^−1^. The Raman spectra were measured using a dispersive Nexus 870 FT-Raman instrument. The instrument is equipped with Nd:YAG laser source operating at 1.064 μm line widths with 200 mW powers. The spectra were recorded with scanning speed of 30 cm^−1^ min^−1^ of spectral width 2 cm^−1^.

### Computational details

All electronic structure calculations were performed using the Gaussian03 suite of programs (Pittsburgh 2003). Geometry optimizations for all compounds and tautomers have been performed using Møller–Plesset perturbation theory (MP2/6-31G(d) and MP2/6-31++G(d,p) methods and DFT at the B3LYP functional in conjunction with the 6-31++G(d,p) and 6-311++G(d,p) basis set. Geometries have been first optimized with full relaxation on the potential energy surfaces at HF/6-31++G(d,p) and HF/6-311++G(d,p) levels. The geometry was then re-optimized at B3LYP/6-31++G(d,p), B3LYP/6-311++G(d,p), (MP2/6-31G(d) and MP2/6-31++G(d,p) levels. For each stationary point, we carried out vibrational frequency calculation at the same level to characterize their nature as minima or transition states and to correct energies for zero-point energy and thermal contribution. The transition states for tautomerization have been located using the eigenvalue-following (EF) optimization procedure as implemented in the Gaussian programs. The nature of the transition states was confirmed by the presence of one negative eigenvalue in the Hessian matrix. The vibrational modes were examined by using the GAUSS-VIEW program (Frisch et al. 2000). In this study two different scaling factors (Sundaraganesan et al. 2005) viz. 0.89 for HF and 0.96 for B3LYP were used to correct the theoretical harmonicity error. Partial charge distributions were calculated using the natural population analysis (NPA) method.

## Additional file

10.1186/s40064-015-1363-2 Optimized geometry of 2A3MP and 2A4MP.
