# Supplementary material for: Tautomerization, molecular structure, transition state structure, and vibrational spectra of 2-aminopyridines: a combined computational and experimental study
Source: Springerplus. 2015 Oct 9;4:586. doi: 10.1186/s40064-015-1363-2 (PMC4628003; doi:10.1186/s40064-015-1363-2)
Supplement: Supplementary file 1 — 10.1186/s40064-015-1363-2 Optimized geometry of 2A3MP and 2A4MP. [file 40064_2015_1363_MOESM1_ESM.docx]

Tautomerization, molecular structure, transition state structure, and vibrational spectra of 2-aminopyridines: A combined computational and experimental study.

Jamelah S. Al-Otaibi

Department of Chemistry, College of Science, Princess Nourah bint Abdulrahman University,

Riyadh 11951, Saudi Arabia

Table S1

Optimized geometrical parameters for 2A3MP molecule (bond lengths in Å, angles in degrees) at two computational methods.

| Parameters | HF | | B3LYP | |
| --- | --- | --- | --- | --- |
|  | 6-31++G(d,p) | 6-311++G(d,p) | 6-31++G(d,p) | 6-311++G(d,p) |
| R(1,2) | 1.377 | 1.374 | 1.392 | 1.388 |
| R(1,6) | 1.411 | 1.410 | 1.420 | 1.417 |
| R(1,9) | 1.508 | 1.508 | 1.507 | 1.505 |
| R(2,3) | 1.394 | 1.393 | 1.400 | 1.397 |
| R(2,7) | 1.077 | 1.077 | 1.088 | 1.086 |
| R(3,4) | 1.376 | 1.374 | 1.392 | 1.388 |
| R(3,10) | 1.074 | 1.074 | 1.085 | 1.083 |
| R(4,5) | 1.326 | 1.325 | 1.340 | 1.337 |
| R(4,11) | 1.076 | 1.077 | 1.088 | 1.086 |
| R(5,6) | 1.317 | 1.315 | 1.34 | 1.336 |
| R(6,8) | 1.380 | 1.381 | 1.387 | 1.386 |
| R(8,12) | 0.996 | 0.995 | 1.011 | 1.010 |
| R(8,13) | 0.994 | 0.994 | 1.009 | 1.008 |
| R(9,14) | 1.086 | 1.087 | 1.097 | 1.096 |
| R(9,15) | 1.088 | 1.089 | 1.099 | 1.097 |
| R(9,16) | 1.084 | 1.084 | 1.094 | 1.092 |
| A(2,1,6) | 116.4 | 116.4 | 116.6 | 116.6 |
| A(2,1,9) | 122.4 | 122.4 | 122.4 | 122.4 |
| A(6,1,9) | 121.2 | 121.2 | 121.0 | 121.0 |
| A(1,2,3) | 120.8 | 120.8 | 120.7 | 120.7 |
| A(1,2,7) | 119.4 | 119.4 | 119.2 | 119.2 |
| A(3,2,7) | 119.8 | 119.8 | 120.2 | 120.1 |
| A(2,3,4) | 117.3 | 117.3 | 117.6 | 117.6 |
| A(2,3,10) | 121.6 | 121.6 | 121.5 | 121.5 |
| A(4,3,10) | 121.1 | 121.1 | 120.9 | 120.9 |
| A(3,4,5) | 123.5 | 123.5 | 123.5 | 123.5 |
| A(3,4,11) | 120.8 | 120.7 | 120.8 | 120.8 |
| A(5,4,11) | 115.8 | 115.8 | 115.7 | 115.8 |
| A(4,5,6) | 118.8 | 118.7 | 118.3 | 118.3 |
| A(1,6,5) | 123.3 | 123.3 | 123.4 | 123.3 |
| A(1,6,8) | 120.5 | 120.4 | 120.8 | 120.7 |
| A(5,6,8) | 116.2 | 116.2 | 115.8 | 115.9 |
| A(6,8,12) | 113.6 | 113.4 | 113.9 | 113.9 |
| A(6,8,13) | 117.5 | 117.1 | 118.2 | 118.1 |
| A(12,8,13) | 114.1 | 113.8 | 114.8 | 114.9 |
| A(1,9,14) | 111.3 | 111.3 | 111.4 | 111.4 |
| A(1,9,15) | 111.9 | 111.8 | 112.0 | 111.9 |
| A(1,9,16) | 110.6 | 110.6 | 110.9 | 110.9 |
| A(14,9,15) | 107.7 | 107.6 | 107.2 | 107.2 |
| A(14,9,16) | 107.9 | 108.0 | 107.9 | 107.9 |
| A(15,9,16) | 107.3 | 107.4 | 107.2 | 107.3 |
| D(6,1,2,3) | -0.3 | -0.3 | -0.4 | -0.3 |
| D(6,1,2,7) | -180.0 | -180.0 | 180.0 | 180.0 |
| D(9,1,2,3) | 179.0 | 179.1 | 179.1 | 179.1 |
| D(9,1,2,7) | -0.6 | -0.6 | -0.6 | -0.6 |
| D(2,1,6,5) | 0.2 | 0.2 | 0.2 | 0.1 |
| D(2,1,6,8) | 177.8 | 177.7 | 177.5 | 177.5 |
| D(9,1,6,5) | -179 | -179.2 | -179.0 | -179.4 |
| D(9,1,6,8) | -1.61 | -1.7 | -2.0 | -1.9 |
| D(2,1,9,14) | -123.0 | -122.7 | -122.0 | -122.0 |
| D(2,1,9,15) | 116.7 | 116.9 | 117.7 | 117.9 |
| D(2,1,9,16) | -2.9 | -2.7 | -2.1 | -1.8 |
| D(6,1,9,14) | 56.5 | 56.6 | 57.2 | 57.4 |
| D(6,1,9,15) | -64.0 | -63.8 | -62.9 | -62.6 |
| D(6,1,9,16) | 176.5 | 176.7 | 177.4 | 177.6 |
| D(1,2,3,4) | 0.1 | 0.1 | 0.1 | 0.1 |
| D(1,2,3,10) | -180.0 | -179.8 | -180.0 | -179.8 |
| D(7,2,3,4) | 179.7 | 179.7 | 179.8 | 179.8 |
| D(7,2,3,10) | -0.2 | -0.2 | -0.2 | -0.1 |
| D(2,3,4,5) | 0.4 | 0.4 | 0.4 | 0.4 |
| D(2,3,4,11) | -180.0 | -179.9 | -180.0 | -180.0 |
| D(10,3,4,5) | -180.0 | -179.8 | -180.0 | -179.7 |
| D(10,3,4,11) | -0.0 | -0.1 | -0.0 | -0.0 |
| D(3,4,5,6) | -0.5 | -0.5 | -0.6 | -0.6 |
| D(11,4,5,6) | 179.7 | 179.8 | 179.7 | 179.7 |
| D(4,5,6,1) | 0.2 | 0.3 | 0.3 | 0.3 |
| D(4,5,6,8) | -177.0 | -177.4 | -177.0 | -177.2 |
| D(1,6,8,12) | 167.6 | 167.2 | 167.8 | 167.7 |
| D(1,6,8,13) | 30.7 | 31.4 | 28.5 | 28.4 |
| D(5,6,8,12) | -14.6 | -15.1 | -14.6 | -14.7 |
| D(5,6,8,13) | -151.0 | -150.9 | -154.0 | -154.0 |

Table S2

Optimized geometrical parameters for 2A4MP molecule (bond lengths in Å, angles in degrees) at two computational methods.

| Parameters | HF | | B3LYP | |
| --- | --- | --- | --- | --- |
|  | 6-31++G(d,p) | 6-311++G(d,p) | 6-31++G(d,p) | 6-311++G(d,p) |
| R(1,2) | 1.377 | 1.374 | 1.391 | 1.387 |
| R(1,6) | 1.405 | 1.404 | 1.412 | 1.409 |
| R(1,9) | 1.076 | 1.075 | 1.087 | 1.085 |
| R(2,3) | 1.402 | 1.400 | 1.408 | 1.405 |
| R(2,7) | 1.508 | 1.507 | 1.509 | 1.507 |
| R(3,4) | 1.375 | 1.373 | 1.390 | 1.387 |
| R(3,10) | 1.075 | 1.075 | 1.086 | 1.084 |
| R(4,5) | 1.328 | 1.327 | 1.342 | 1.338 |
| R(4,11) | 1.077 | 1.077 | 1.088 | 1.087 |
| R(5,6) | 1.317 | 1.315 | 1.340 | 1.337 |
| R(6,8) | 1.376 | 1.378 | 1.385 | 1.384 |
| R(7,12) | 1.086 | 1.086 | 1.096 | 1.094 |
| R(7,13) | 1.085 | 1.086 | 1.096 | 1.094 |
| R(7,14) | 1.083 | 1.083 | 1.094 | 1.092 |
| R(8,15) | 0.995 | 0.995 | 1.011 | 1.009 |
| R(8,16) | 0.994 | 0.994 | 1.009 | 1.008 |
| A(2,1,6) | 119.1 | 119.1 | 119.4 | 119.5 |
| A(2,1,9) | 121 | 121 | 120.5 | 120.5 |
| A(6,1,9) | 119.8 | 119.8 | 120.0 | 120.0 |
| A(1,2,3) | 117.9 | 117.9 | 117.7 | 117.7 |
| A(1,2,7) | 121.5 | 121.5 | 121.4 | 121.4 |
| A(3,2,7) | 120.6 | 120.6 | 120.9 | 120.9 |
| A(2,3,4) | 118.2 | 118.2 | 118.6 | 118.6 |
| A(2,3,10) | 121.3 | 121.3 | 121.1 | 121.1 |
| A(4,3,10) | 120.5 | 120.5 | 120.4 | 120.4 |
| A(3,4,5) | 124.4 | 124.4 | 124.3 | 124.2 |
| A(3,4,11) | 120.2 | 120.1 | 120.3 | 120.2 |
| A(5,4,11) | 115.5 | 115.5 | 115.5 | 115.5 |
| A(4,5,6) | 117.7 | 117.6 | 117.3 | 117.4 |
| A(1,6,5) | 122.8 | 122.8 | 122.7 | 122.7 |
| A(1,6,8) | 120.5 | 120.4 | 121.0 | 121.0 |
| A(5,6,8) | 116.7 | 116.8 | 116.2 | 116.3 |
| A(2,7,12) | 110.5 | 110.4 | 110.9 | 110.9 |
| A(2,7,13) | 110.5 | 110.4 | 110.9 | 110.8 |
| A(2,7,14) | 111.5 | 111.5 | 111.6 | 111.6 |
| A(12,7,13) | 107.6 | 107.6 | 107.1 | 107.2 |
| A(12,7,14) | 108.3 | 108.4 | 108.1 | 108.1 |
| A(13,7,14) | 108.3 | 108.4 | 108.0 | 108.1 |
| A(6,8,15) | 114.4 | 114.1 | 114.5 | 114.5 |
| A(6,8,16) | 117.2 | 116.8 | 117.8 | 117.7 |
| A(15,8,16) | 114.7 | 114.4 | 115.2 | 115.1 |
| D(6,1,2,3) | 0.186 | 0.2 | 0.2 | 0.2 |
| D(6,1,2,7) | 180 | -180.0 | -180.0 | -179.9 |
| D(9,1,2,3) | -179 | -179.0 | -179.0 | -179.2 |
| D(9,1,2,7) | 0.689 | 0.6 | 0.6 | 0.6 |
| D(2,1,6,5) | 0.029 | 0.01 | 0.0 | 0.0 |
| D(2,1,6,8) | -178.0 | -178.0 | -178.0 | -177.7 |
| D(9,1,6,5) | 179.3 | 179.4 | 179.5 | 179.5 |
| D(9,1,6,8) | 1.4 | 1.4 | 1.8 | 1.8 |
| D(1,2,3,4) | -0.1 | -0.1 | -0.1 | -0.1 |
| D(1,2,3,10) | 179.9 | 179.9 | 179.9 | 179.9 |
| D(7,2,3,4) | -180.0 | -180.0 | -180.0 | -180.0 |
| D(7,2,3,10) | 0.1 | 0.1 | 0.1 | 0.1 |
| D(1,2,7,12) | 120.5 | 120.7 | 120.8 | 120.8 |
| D(1,2,7,13) | -120 | -120 | -120 | -120.3 |
| D(1,2,7,14) | 0.023 | 0.2 | 0.2 | 0.2 |
| D(3,2,7,12) | -59.7 | -59.5 | -59.4 | -59.4 |
| D(3,2,7,13) | 59.34 | 59.4 | 59.5 | 59.5 |
| D(3,2,7,14) | 179.8 | 179.9 | -180.0 | -180.0 |
| D(2,3,4,5) | -0.21 | -0.2 | -0.2 | -0.2 |
| D(2,3,4,11) | -180.0 | -180.0 | -180.0 | -179.9 |
| D(10,3,4,5) | 179.8 | 179.8 | 179.7 | 179.7 |
| D(10,3,4,11) | 0.051 | 0.1 | 0.0 | 0.0 |
| D(3,4,5,6) | 0.422 | 0.4 | 0.5 | 0.5 |
| D(11,4,5,6) | -180.0 | -180.0 | -180.0 | -179.8 |
| D(4,5,6,1) | -0.33 | -0.3 | -0.4 | -0.3 |
| D(4,5,6,8) | 177.7 | 177.7 | 177.4 | 177.4 |
| D(1,6,8,15) | -166.0 | -165.0 | -166.0 | -166.1 |
| D(1,6,8,16) | -27 | -27.8 | -25.9 | -26.0 |
| D(5,6,8,15) | 16.37 | 16.9 | 16.1 | 16.1 |
| D(5,6,8,16) | 154.9 | 154.1 | 156.3 | 156.2 |

Table S3

Optimized geometrical parameters for 2A3MP molecule (bond lengths in Å, angles in degrees) using Møller–Plesset perturbation theory (MP2/6-31G(d) and MP2/6-31++G(d,p) methods.

| Parameters | MP2 | |
| --- | --- | --- |
|  | 6-31G(d) | 6-31++G(d,p) |
| R(1,2) | 1.392 | 1.394 |
| R(1,6) | 1.413 | 1.414 |
| R(1,9) | 1.502 | 1.502 |
| R(2,3) | 1.397 | 1.399 |
| R(2,7) | 1.089 | 1.085 |
| R(3,4) | 1.390 | 1.392 |
| R(3,10) | 1.086 | 1.082 |
| R(4,5) | 1.346 | 1.347 |
| R(4,11) | 1.089 | 1.084 |
| R(5,6) | 1.342 | 1.343 |
| R(6,8) | 1.397 | 1.396 |
| R(8,12) | 1.016 | 1.012 |
| R(8,13) | 1.014 | 1.010 |
| R(9,14) | 1.095 | 1.092 |
| R(9,15) | 1.097 | 1.094 |
| R(9,16) | 1.093 | 1.089 |
| A(2,1,6) | 116.9 | 116.9 |
| A(2,1,9) | 122.7 | 122.5 |
| A(6,1,9) | 120.4 | 120.6 |
| A(1,2,3) | 120.2 | 120.2 |
| A(1,2,7) | 119.4 | 119.3 |
| A(3,2,7) | 120.4 | 120.4 |
| A(2,3,4) | 118.1 | 118.0 |
| A(2,3,10) | 121.3 | 121.4 |
| A(4,3,10) | 120.6 | 120.6 |
| A(3,4,5) | 123.5 | 123.4 |
| A(3,4,11) | 121.0 | 121.1 |
| A(5,4,11) | 115.5 | 115.6 |
| A(4,5,6) | 117.6 | 117.7 |
| A(1,6,5) | 123.8 | 123.7 |
| A(1,6,8) | 120.2 | 120.3 |
| A(5,6,8) | 115.9 | 115.9 |
| A(6,8,12) | 110.7 | 111.8 |
| A(6,8,13) | 115.0 | 115.7 |
| A(12,8,13) | 111.8 | 112.5 |
| A(1,9,14) | 110.6 | 110.7 |
| A(1,9,15) | 111.7 | 111.6 |
| A(1,9,16) | 110.8 | 110.6 |
| A(14,9,15) | 107.5 | 107.7 |
| A(14,9,16) | 108.6 | 108.4 |
| A(15,9,16) | 107.6 | 107.7 |
| D(6,1,2,3) | -1.2 | -1.4 |
| D(6,1,2,7) | 179.7 | 179.7 |
| D(9,1,2,3) | 178.6 | 178.9 |
| D(9,1,2,7) | -0.4 | 0.0 |
| D(2,1,6,5) | 1.255 | 1.4 |
| D(2,1,6,8) | 176.8 | 176.6 |
| D(9,1,6,5) | -178.6 | -179.0 |
| D(9,1,6,8) | -3.0 | -3.7 |
| D(2,1,9,14) | -126.6 | -126.0 |
| D(2,1,9,15) | 113.7 | 114.1 |
| D(2,1,9,16) | -6.2 | -5.8 |
| D(6,1,9,14) | 53.2 | 54.3 |
| D(6,1,9,15) | -66.5 | -65.7 |
| D(6,1,9,16) | 173.7 | 174.5 |
| D(1,2,3,4) | 0.4 | 0.6 |
| D(1,2,3,10) | -179.6 | -180.0 |
| D(7,2,3,4) | 179.4 | 179.5 |
| D(7,2,3,10) | -0.6 | -0.7 |
| D(2,3,4,5) | 0.6 | 0.3 |
| D(2,3,4,11) | -179.8 | -180.0 |
| D(10,3,4,5) | -179.5 | -180.0 |
| D(10,3,4,11) | 0.2 | 0.3 |
| D(3,4,5,6) | -0.6 | -0.3 |
| D(11,4,5,6) | 179.7 | 179.9 |
| D(4,5,6,1) | -0.3 | -0.6 |
| D(4,5,6,8) | -176.1 | -176.0 |
| D(1,6,8,12) | 168.7 | 167.7 |
| D(1,6,8,13) | 40.8 | 37.1 |
| D(5,6,8,12) | -15.4 | -16.8 |

Table S4

Optimized geometrical parameters for 2A4MP molecule (bond lengths in Å, angles in degrees) using Møller–Plesset perturbation theory (MP2/6-31G(d) and MP2/6-31++G(d,p) methods.

| Parameters | MP2 | |
| --- | --- | --- |
|  | 6-31G(d) | 6-31++G(d,p) |
| R(1,2) | 1.390 | 1.392 |
| R(1,6) | 1.406 | 1.407 |
| R(1,9) | 1.089 | 1.085 |
| R(2,3) | 1.403 | 1.404 |
| R(2,7) | 1.505 | 1.505 |
| R(3,4) | 1.389 | 1.391 |
| R(3,10) | 1.087 | 1.083 |
| R(4,5) | 1.347 | 1.348 |
| R(4,11) | 1.089 | 1.084 |
| R(5,6) | 1.342 | 1.343 |
| R(6,8) | 1.395 | 1.395 |
| R(7,12) | 1.095 | 1.092 |
| R(7,13) | 1.094 | 1.090 |
| R(7,14) | 1.093 | 1.090 |
| R(8,15) | 1.015 | 1.011 |
| R(8,16) | 1.014 | 1.010 |
| A(2,1,6) | 119.5 | 119.6 |
| A(2,1,9) | 120.5 | 120.5 |
| A(6,1,9) | 119.9 | 119.9 |
| A(1,2,3) | 117.6 | 117.6 |
| A(1,2,7) | 121.4 | 121.2 |
| A(3,2,7) | 121.0 | 121.2 |
| A(2,3,4) | 118.9 | 118.9 |
| A(2,3,10) | 120.8 | 120.9 |
| A(4,3,10) | 120.3 | 120.2 |
| A(3,4,5) | 124.1 | 124.0 |
| A(3,4,11) | 120.6 | 120.6 |
| A(5,4,11) | 115.3 | 115.4 |
| A(4,5,6) | 116.9 | 117.0 |
| A(1,6,5) | 123.0 | 123.0 |
| A(1,6,8) | 120.9 | 120.8 |
| A(5,6,8) | 116.0 | 116.1 |
| A(2,7,12) | 110.9 | 110.8 |
| A(2,7,13) | 110.9 | 110.8 |
| A(2,7,14) | 111.2 | 111.0 |
| A(12,7,13) | 107.5 | 107.8 |
| A(12,7,14) | 108.0 | 108 |
| A(13,7,14) | 108.2 | 108.3 |
| A(6,8,15) | 111.4 | 112.4 |
| A(6,8,16) | 114.9 | 115.4 |
| D(6,1,2,3) | 0.7 | 112.8 |
| D(6,1,2,7) | -179.0 | 0.8 |
| D(9,1,2,3) | -179.0 | -178.0 |
| D(9,1,2,7) | 1.9 | -179.0 |
| D(2,1,6,5) | -0.5 | 2.3 |
| D(2,1,6,8) | -177.0 | -0.6 |
| D(9,1,6,5) | 178.9 | -176.0 |
| D(9,1,6,8) | 2.8 | 178.9 |
| D(1,2,3,4) | -0.3 | 3.0 |
| D(1,2,3,10) | 179.6 | -0.5 |
| D(7,2,3,4) | 179.0 | 179.5 |
| D(7,2,3,10) | -1.1 | 178.5 |
| D(1,2,7,12) | 106.7 | -1.4 |
| D(1,2,7,13) | -134.0 | 95.8 |
| D(1,2,7,14) | -13.4 | -145.0 |
| D(3,2,7,12) | -72.5 | -24.2 |
| D(3,2,7,13) | 46.9 | -83.2 |
| D(3,2,7,14) | 167.4 | 36.4 |
| D(2,3,4,5) | -0.4 | 156.8 |
| D(2,3,4,11) | 179.9 | -0.1 |
| D(10,3,4,5) | 179.7 | -180.0 |
| D(10,3,4,11) | 0.0 | 179.9 |
| D(3,4,5,6) | 0.5 | -0.1 |
| D(11,4,5,6) | -180.0 | 0.3 |
| D(4,5,6,1) | -0.1 | -180.0 |
| D(4,5,6,8) | 176.3 | 0.0 |
| D(1,6,8,15) | -165.0 | 176.0 |
| D(1,6,8,16) | -36.0 | -165.0 |
| D(5,6,8,15) | 18.6 | -33.9 |
| D(5,6,8,16) | 147.6 | 18.8 |

Table S5

Optimized geometrical parameters of transition state structures for 2A4MP molecule (bond lengths in Å , angles in degrees) using B3LYP/6-311++G(d,p) method.

| Proton transfer TS | | Inversion at N TS | |
| --- | --- | --- | --- |
| R(1,2) | 1.387 | R(1,2) | 1.386 |
| R(1,6) | 1.415 | R(1,6) | 1.411 |
| R(1,9) | 1.083 | R(1,9) | 1.085 |
| R(2,3) | 1.417 | R(2,3) | 1.405 |
| R(2,7) | 1.507 | R(2,7) | 1.507 |
| R(3,4) | 1.384 | R(3,4) | 1.387 |
| R(3,10) | 1.082 | R(3,10) | 1.084 |
| R(4,5) | 1.333 | R(4,5) | 1.338 |
| R(4,11) | 1.084 | R(4,11) | 1.087 |
| R(5,6) | 1.375 | R(5,6) | 1.338 |
| R(5,15) | 1.306 | R(6,8) | 1.371 |
| R(6,8) | 1.328 | R(7,12) | 1.094 |
| R(6,15) | 1.695 | R(7,13) | 1.094 |
| R(7,12) | 1.094 | R(7,14) | 1.092 |
| R(7,13) | 1.094 | R(8,15) | 1.006 |
| R(7,14) | 1.091 | R(8,16) | 1.003 |
| R(8,15) | 1.395 | A(2,1,6) | 119.4 |
| R(8,16) | 1.01 | A(2,1,9) | 120.5 |
| A(2,1,6) | 118.4 | A(6,1,9) | 120.0 |
| A(2,1,9) | 121.1 | A(1,2,3) | 117.8 |
| A(6,1,9) | 120.5 | A(1,2,7) | 121.3 |
| A(1,2,3) | 120.1 | A(3,2,7) | 120.9 |
| A(1,2,7) | 120.6 | A(2,3,4) | 118.5 |
| A(3,2,7) | 119.3 | A(2,3,10) | 121.1 |
| A(2,3,4) | 119.2 | A(4,3,10) | 120.4 |
| A(2,3,10) | 120.8 | A(3,4,5) | 124.4 |
| A(4,3,10) | 120.1 | A(3,4,11) | 120.2 |
| A(3,4,5) | 120.4 | A(5,4,11) | 115.5 |
| A(3,4,11) | 122.4 | A(4,5,6) | 117.3 |
| A(5,4,11) | 117.2 | A(1,6,5) | 122.6 |
| A(4,5,6) | 122.4 | A(1,6,8) | 121.2 |
| A(4,5,15) | 159.2 | A(5,6,8) | 116.2 |
| A(1,6,5) | 119.5 | A(2,7,12) | 110.8 |
| A(1,6,8) | 138.3 | A(2,7,13) | 110.8 |
| A(1,6,15) | 168.4 | A(2,7,14) | 111.6 |
| A(5,6,8) | 102.3 | A(12,7,13) | 107.2 |
| A(2,7,12) | 110.7 | A(12,7,14) | 108.1 |
| A(2,7,13) | 110.7 | A(13,7,14) | 108.1 |
| A(2,7,14) | 111.7 | A(6,8,15) | 118.4 |
| A(12,7,13) | 107.1 | A(6,8,16) | 122.0 |
| A(12,7,14) | 108.3 | A(15,8,16) | 119.6 |
| A(13,7,14) | 108.3 | D(6,1,2,3) | -0.0 |
| A(6,8,16) | 123.1 | D(6,1,2,7) | 180.0 |
| A(5,15,8) | 102.4 | D(9,1,2,3) | 180.0 |
| D(6,1,2,3) | -0.0 | D(9,1,2,7) | -0.0 |
| D(6,1,2,7) | 180.0 | D(2,1,6,5) | 0.0 |
| D(9,1,2,3) | -180.0 | D(2,1,6,8) | 180.0 |
| D(9,1,2,7) | -0.0 | D(9,1,6,5) | 180.0 |
| D(2,1,6,5) | -0.0 | D(9,1,6,8) | 0.0 |
| D(2,1,6,8) | -180.0 | D(1,2,3,4) | 0.0 |
| D(2,1,6,15) | 0.0 | D(1,2,3,10) | -180.0 |
| D(9,1,6,5) | 180.0 | D(7,2,3,4) | -180.0 |
| D(9,1,6,8) | 0.0 | D(7,2,3,10) | 0.0 |
| D(9,1,6,15) | 180.0 | D(1,2,7,12) | 120.6 |
| D(1,2,3,4) | 0.0 | D(1,2,7,13) | -121.0 |
| D(1,2,3,10) | -180.0 | D(1,2,7,14) | 0.0 |
| D(7,2,3,4) | -180.0 | D(3,2,7,12) | -59.4 |
| D(7,2,3,10) | 0.0 | D(3,2,7,13) | 59.4 |
| D(1,2,7,12) | 120.7 | D(3,2,7,14) | -180.0 |
| D(1,2,7,13) | -121.0 | D(2,3,4,5) | 0.0 |
| D(1,2,7,14) | 0.0 | D(2,3,4,11) | 180.0 |
| D(3,2,7,12) | -59.3 | D(10,3,4,5) | 180.0 |
| D(3,2,7,13) | 59.3 | D(10,3,4,11) | 0.0 |
| D(3,2,7,14) | -180.0 | D(3,4,5,6) | 0.0 |
| D(2,3,4,5) | -0.0 | D(11,4,5,6) | 180.0 |
| D(2,3,4,11) | -180.0 | D(4,5,6,1) | 0.0 |
| D(10,3,4,5) | 180.0 | D(4,5,6,8) | -180.0 |
| D(10,3,4,11) | 0.00 | D(1,6,8,15) | 180.0 |
| D(3,4,5,6) | 0.0 | D(1,6,8,16) | 0.0 |
| D(3,4,5,15) | 180.0 | D(5,6,8,15) | 0.0 |
| D(11,4,5,6) | 180.0 | D(5,6,8,16) | 180.0 |
| D(11,4,5,15) | 0.0 |  |  |
| D(4,5,6,1) | 0.0 |  |  |
| D(4,5,6,8) | 180.0 |  |  |
| D(4,5,15,8) | -180.0 |  |  |
| D(1,6,8,16) | 0.0 |  |  |
| D(5,6,8,16) | 180.0 |  |  |

Table S6

Wiberg bond index matrix in the natural atomic orbital basis (NAO) for 2A4MP1 computed at B3LYP/6-311+G(d,p).

| Atom | C1 | C2 | C3 | C4 | N5 | C6 | C7 | N8 | H9 | H10 | H11 | H12 | H13 | H14 | H15 | H16 |
| --- | --- | --- | --- | --- | --- | --- | --- | --- | --- | --- | --- | --- | --- | --- | --- | --- |
| C1 | 0.000 | 1.458 | 0.016 | 0.104 | 0.030 | 1.307 | 0.011 | 0.048 | 0.925 | 0.010 | 0.001 | 0.014 | 0.014 | 0.002 | 0.011 | 0.002 |
| C2 | 1.458 | 0.000 | 1.347 | 0.016 | 0.087 | 0.016 | 1.036 | 0.013 | 0.005 | 0.004 | 0.008 | 0.003 | 0.003 | 0.002 | 0.000 | 0.000 |
| C3 | 0.016 | 1.347 | 0.000 | 1.463 | 0.036 | 0.085 | 0.011 | 0.036 | 0.011 | 0.925 | 0.003 | 0.005 | 0.005 | 0.008 | 0.001 | 0.001 |
| C4 | 0.104 | 0.016 | 1.463 | 0.000 | 1.397 | 0.013 | 0.010 | 0.010 | 0.001 | 0.004 | 0.925 | 0.000 | 0.000 | 0.000 | 0.000 | 0.001 |
| N5 | 0.030 | 0.087 | 0.036 | 1.397 | 0.000 | 1.369 | 0.002 | 0.076 | 0.011 | 0.010 | 0.019 | 0.004 | 0.004 | 0.000 | 0.005 | 0.011 |
| C6 | 1.307 | 0.016 | 0.085 | 0.013 | 1.369 | 0.000 | 0.009 | 1.145 | 0.003 | 0.001 | 0.009 | 0.000 | 0.000 | 0.000 | 0.002 | 0.002 |
| C7 | 0.011 | 1.036 | 0.011 | 0.010 | 0.002 | 0.009 | 0.000 | 0.000 | 0.003 | 0.002 | 0.001 | 0.927 | 0.927 | 0.945 | 0.000 | 0.000 |
| N8 | 0.048 | 0.013 | 0.036 | 0.010 | 0.076 | 1.145 | 0.000 | 0.000 | 0.001 | 0.001 | 0.000 | 0.001 | 0.001 | 0.000 | 0.831 | 0.844 |
| H9 | 0.925 | 0.005 | 0.011 | 0.001 | 0.011 | 0.003 | 0.003 | 0.001 | 0.000 | 0.000 | 0.001 | 0.000 | 0.000 | 0.001 | 0.000 | 0.000 |
| H10 | 0.010 | 0.004 | 0.925 | 0.004 | 0.010 | 0.001 | 0.002 | 0.001 | 0.000 | 0.000 | 0.002 | 0.000 | 0.000 | 0.000 | 0.000 | 0.000 |
| H11 | 0.001 | 0.008 | 0.003 | 0.925 | 0.019 | 0.009 | 0.001 | 0.000 | 0.001 | 0.002 | 0.000 | 0.000 | 0.000 | 0.000 | 0.000 | 0.000 |
| H12 | 0.014 | 0.003 | 0.005 | 0.000 | 0.004 | 0.000 | 0.927 | 0.001 | 0.000 | 0.000 | 0.000 | 0.000 | 0.001 | 0.001 | 0.000 | 0.000 |
| H13 | 0.014 | 0.003 | 0.005 | 0.000 | 0.004 | 0.000 | 0.927 | 0.001 | 0.000 | 0.000 | 0.000 | 0.001 | 0.000 | 0.001 | 0.000 | 0.000 |
| H14 | 0.002 | 0.002 | 0.008 | 0.000 | 0.000 | 0.000 | 0.945 | 0.000 | 0.001 | 0.000 | 0.000 | 0.001 | 0.001 | 0.000 | 0.000 | 0.000 |
| H15 | 0.011 | 0.000 | 0.001 | 0.000 | 0.005 | 0.002 | 0.000 | 0.831 | 0.000 | 0.000 | 0.000 | 0.000 | 0.000 | 0.000 | 0.000 | 0.000 |
| H16 | 0.002 | 0.000 | 0.001 | 0.001 | 0.011 | 0.002 | 0.000 | 0.844 | 0.000 | 0.000 | 0.000 | 0.000 | 0.000 | 0.000 | 0.000 | 0.000 |
